# Fertility Preservation in Iranian Cancer Patients: A Continuing Neglect

**DOI:** 10.22074/ijfs.2017.4960

**Published:** 2017-09-03

**Authors:** Gholamreza Toogeh, Mohammadreza Razzaghof, Fariba Zarrabi

**Affiliations:** 1 Department of Internal Medicine, School of Medicine, Tehran University of Medical Sciences, Tehran, Iran; 2 Thrombosis Hemostasis Research Center, Tehran University of Medical Sciences, Tehran, Iran

It is not to be denied that one of the greatest breakthroughs of modern medicine is the day-today improvement in the diagnosis and treatment of cancer. Global statistics show a declining rate of mortality from cancer and rising rate of survival from this ominous disease (1). Mortality data over the past quarter-century is quite promising as it shows a decreasing mortality rate from all cancers combined by 1.5% per year since 1993 in men and by 0.8% per year since 1992 in women (2). It is most fortunate which all of the most common cancers in men (lung, colorectal, and prostate) and women (breast and colorectal) show this decreasing trend. Even lung cancer mortality in women has finally leveled off after several decades of increase. Despite such improvements in the survival rate of cancers, the incidence rate in Iran shows an increasing trend (3-5).

Iran, as a developing country, is undergoing an epidemiologic transition from communicable to non-communicable diseases (6). Breast cancer, the most common malignancy in women, has shown an increasing incidence in Iran in recent decades, especially in women of reproductive age (7, 8). The largest age group of Iranian women with breast cancer is among those 40-49 years of age. Although worldwide, breast cancer is uncommon in women less than 40 years of age, 23% of female breast cancer cases in Iran are under the age of 40 years (8). Thus, compared with the global average, the incidence of breast cancer in Iran is nearly one decade behind (9). A total of 42% of cervical cancer cases are diagnosed in women less than 45 years of age (10). In colorectal cancer, 42.9% of patients are younger than 50 years (11). Therefore, it appears that a considerable group of our cancer patients are or will be of reproductive or pre-pubertal age in the future.

## Detrimental effects of cancer on fertility and mental health

It should be noted that cancer does not bequeath a valuable heritage to its survivors; rather, there are considerable prolonged physical and mental complications. One of the most important is the detrimental effect of cancer on fertility and reproduction in survivors. Fertility in patients with cancer can be impaired in one of two ways, either as a sequel of the cancer itself or an adverse effect of the treatment protocol in use such as radio-chemotherapy regimens or bone marrow transplantations (12). In Iran, the increasing incidence of cancer, improving trend in survival rates, and significant proportion of young patients with cancer attach considerable importance to this issue. Delaying childbearing for social and financial reasons causes even more women to endure fertility threats because of earlystage cancer diagnoses (13). Infertility that results from cancer or its treatment jeopardizes self-esteem, personal identity, sexuality, and self-image of cancer patients. It also causes feelings of emptiness and defeat, and a negative effect on families and marriages (14).

## Available fertility preservation options

**Fig.1:**
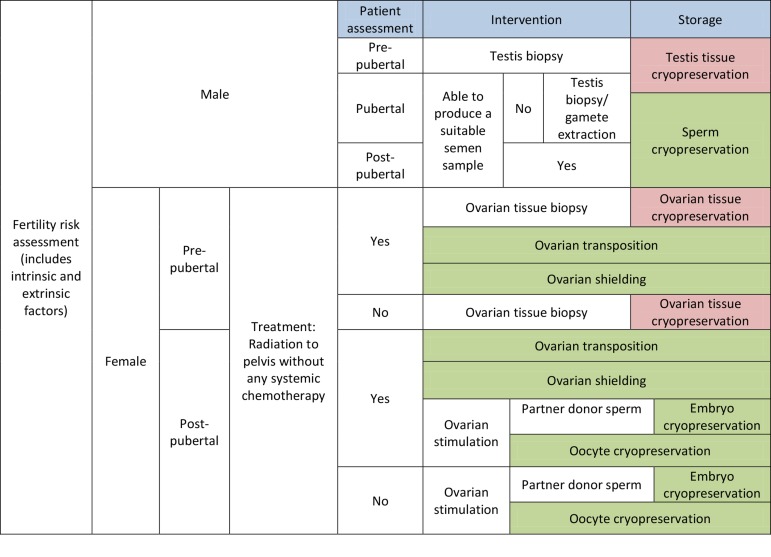
Fertility preservation options for both male and female with cancer.

Fertility preservation options in male patients include sperm collection either by masturbation, electroejaculation, or testicular biopsy followed by cryopreservation of semen and testis tissue cryopreservation (15). In women, due to the nonreplenishable number of ovarian follicles, fertility preservation is more complex and depends on patient age, urgency of the treatment, and the regimen and treatment dosages. These techniques include immature and mature oocyte cryopreservation, ovarian tissue cryopreservation, ovarian suppression with a gonadotropin-releasing hormone (GnRH) agonist, ovarian transposition, embryo cryopreservation, gonadal shielding, and conservative gynecologic surgery (16, 17). According to American Society of Clinical Oncology (ASCO) and European Society for Medical Oncology (ESMO) guidelines on fertility preservation for cancer patients, established, highly recommended fertility preservation methods include sperm cryopreservation in males and embryo and oocyte cryopreservation in females. Patients should also be informed that other methods (i.e., testicular or ovarian tissue cryopreservation) are experimental. Hormone therapy to preserve fertility should not be recommended in males or females, as there is insufficient evidence of its effectiveness (18, 19). Figure 1 summarizes the fertility preservation options for male and female patients. Of note, these fertility preservation methods are available, in various forms, in Iran (20-22).

## Lack of knowledge: physicians versus patients

It is a fact that women with cancers report great emotional distress and misgiving from unmet information about fertility preservation options besides cancer treatment (16, 23, 24). Ghorbani et al. (25) studied Iranian oncologists’ attitudes on fertility preservation. Only 46% of oncologists expressed awareness of fertility preservation techniques. Although the oncologists believed that radio-chemotherapy had a 30% damage rate on reproductive organs, 67% of them believed that fertility preservation should be offered to all patients. Howeveronly 40% offered fertility preservation. Of note, only 15% of oncologists delayed treatment to refer patients to fertility preservation centers. The most important reason why parents of children with cancer did not think of fertility preservation before cancer treatment was the lack of knowledge. Sadri-Ardekani et al. (26) studied on parental attitudes toward fertility preservation in 456 boys with cancer. They reported that parents of boys with cancer had limited knowledge about the risks of infertility due to cancer treatment. However, the majority desired some sort of fertility preservation once informed about these risks. More than one-third of parents wanted some sort of fertility preservation even if the chance of infertility was less than 20%.

In sum, the results of these studies highlight the fact that knowledge of both oncologists and patients about the necessity and importance of fertility preservation in Iran is inadequate. The increasing incidence of cancer, improving trend in survival rates, and significant proportion of young patients with cancer in Iran emphasize that this important issue, termed "oncofertility" by Dr. Teresa Woodruff in 2006 as new interdisciplinary field of obstetrics and gynecology (16), should be brought to the fore front in the health system policies of Iran. In order to achieve this, we make the following recommendations:

1. Ministry of Health and Medical Education organized and supervised educational programs, panels, and seminars should be held with the contribution of all related medical subspecialties including adult and pediatric oncologists, gynecologists, surgical oncologists, urologists, radiotherapists, and embryologists.
2. Regulations should be established by deputies of treatment in medical universities to oblige fertility counseling before the start of cancer treatment, in the same manner as routine laboratory tests and cardiology counseling.
3. National clinical guidelines should be developed for proper case selection and referral, and the choice of an appropriate fertility preservation technique. These guidelines should be developed by a committee of relevant specialist groups and supervised by the Treatment Deputy of the Ministry of Health and Medical Education.
4. Standard institutes specialized in the preparation and preservation of reproductive tissues that include sperm, ovule, fetus, and testis and ovary tissues should be endorsed, equipped and expanded under the supervision of the Ministry of Health and Medical Education.
5. Appropriate insurance and financial support should be provided for adequate coverage of costs, guaranteeing the integrity of tissues and compensation for probable damage.
